# Multiple HPV Infections and Viral Load Association in Persistent Cervical Lesions in Mexican Women

**DOI:** 10.3390/v12040380

**Published:** 2020-03-31

**Authors:** Mariel A. Oyervides-Muñoz, Antonio A. Pérez-Maya, Celia N. Sánchez-Domínguez, Anais Berlanga-Garza, Mauro Antonio-Macedo, Lezmes D. Valdéz-Chapa, Ricardo M. Cerda-Flores, Victor Trevino, Hugo A. Barrera-Saldaña, María L. Garza-Rodríguez

**Affiliations:** 1 Departamento de Bioquímica y Medicina Molecular, Facultad de Medicina, Universidad Autónoma de Nuevo León, Av. Francisco I. Madero S/N, Mitras Centro Monterrey, 64460 Nuevo León, Mexico; mariel.oyervidesmnz@uanl.edu.mx (M.A.O.-M.); bioquimicomty@gmail.com (A.A.P.-M.); celianohemi@hotmail.com (C.N.S.-D.); 2 Departamento de Ginecología y Obstetricia, Hospital Universitario “Dr. José Eleuterio González”, Universidad Autónoma de Nuevo León, Av. Francisco I. Madero S/N, Mitras Centro, 64460 Nuevo León, Mexico; ana_bg88@hotmail.com (A.B.-G.); dr.mauro8207@gmail.com (M.A.-M.); dr.lezmes@gmail.com (L.D.V.-C.); 3 Facultad de Enfermería, Universidad Autónoma de Nuevo León, Av. Dr. José Eleuterio González 1500, Mitras Centro, 64460 Nuevo León, Mexico; ricardocerda_mx@yahoo.com.mx; 4 Tecnologico de Monterrey, Escuela de Medicina y Ciencias de la Salud, 3000 Av. Morones Prieto, Colonia Los Doctores, 64710 Nuevo León, Mexico; vtrevino@tec.mx; 5 Vitagénesis SA. Blvd. Puerta del Sol 1005. Colinas de San Jerónimo. Monterrey, 64630 Nuevo León, Mexico; 6 Centro Universitario contra el Cancer, Hospital Universitario “Dr. José Eleuterio González”, Universidad Autónoma de Nuevo León, Av. Francisco I. Madero S/N, Mitras Centro, 64460 Nuevo León, Mexico

**Keywords:** HPV types, multiple HPV infection, persistence, HPV typing, HPV viral load

## Abstract

Persistent high-risk human papillomavirus (HR-HPV) infections play a major role in the development of invasive cervical cancer (CC), and screening for such infections is in many countries the primary method of detecting and preventing CC. HPV typing can be used for triage and risk stratification of women with atypical squamous cells of undetermined significance (ASC-US)/low-grade cervical lesions (LSIL), though the current clinical practice in Mexico is to diagnose CC or its preceding conditions mainly via histology and HR-HPV detection. Additional information regarding these HPV infections, such as viral load and co-infecting agents, might also be useful for diagnosing, predicting, and evaluating the possible consequences of the infection and of its prevention by vaccination. The goal of this follow-up hospital case study was to determine if HPV types, multiple HPV infections, and viral loads were associated with infection persistence and the cervical lesion grade. A total of 294 cervical cytology samples drawn from patients with gynecological alterations were used in this study. HPV types were identified by real-time PCR DNA analysis. A subset of HPV-positive patients was reevaluated to identify persistent infections. We identified HPV types 16, 18, and 39 as the most prevalent. One hundred five of the patients (59%) were infected with more than one type of HPV. The types of HPV associated with multiple HPV infections were 16, 18, and 39. In the follow-up samples, 38% of patients had not cleared the initially detected HPV infection, and these were considered persistent. We found here an association between multiple HPV infections and high viral loads with and infection persistence. Our findings suggest there are benefits in ascertaining viral load and multiple HPV infections status of HR-HPV infections for predicting the risk of persistence, a requirement for developing CC. These findings contribute to our understanding of HPV epidemiology and may allow screening programs to better assess the cancer-developing risks associated with individual HR-HPV infections.

## 1. Introduction

Although the USA is on track to eliminate cervical cancer (CC) as a public health problem within the next two to three decades, similar progress in Latin American countries has been absent [1]. In Mexico, multiple barriers prevent a similar reduction in CC mortality: low educational levels, illiteracy in the adult population, low coverage of HPV vaccination, and cultural barriers [2]. According to the Mexican Census of Population and Housing, the mortality rate of CC decreased from 10.2 to 9.7 from 1990 to 2010 [3]. This reduction in mortality was mainly attributable to a decreased in birth rate and an increase in cervical cytology coverage [4]. In 2018 the crude mortality rate of CC was 6.3 [5]. Rural zones still show the highest incidence and mortality rates, even though CC screening coverage has increased from 33.3% in 2000 to 49% in 2012 [3].

The infection persistence of high-risk (HR) human papillomavirus (HPV) plays a major role in CC [6], the second most common cancer type for women in Mexico [7]. Most HPV infections are transient (70%-80%) and cleared in less than 2 years. Genetic susceptibility, immune function, and environmental factors are all putative effectors of viral clearance [8]. Of all HPV infections, 20%-30% are persistent, and 1%-2% lead to CC development [9]. 

The most common HR-HPV types in CC (16, 18, 31, 33, 35, 39, 45, 51, 52, 56, 58, 59 and 68) are associated with low-grade cervical lesions (LSIL), high-grade cervical lesions (HSIL), and CC [10]. HPV types 16 and 18 are responsible for 70% of HPV associated cancer cases worldwide [11]. In Latin America, the joint prevalence of HPV 16 and/or 18 is 4.7% in normal cytology, 15% in LSIL, 41% in HSIL, and 63% in CC [12]. A recent study reported that HPV types 16 and/or 18 have been found in 46.5% and 8.9% of HSIL samples from Latin American and Caribbean populations, respectively [13]. 

In Mexico, the prevalence of different HPV types in screened women varies by region. In the northeast, the types 59, 52, and 16 are the most commonly encountered [14]; in the southeast, the types 16 and 18, the most dangerous in terms of developing CC, are the most common [15]; and in the western regions, the types 16, 18, and 58 are the most prevalent in the population [16,17]. 

By 2009, 12 molecular biology laboratories for HR-HPV detection were operating in Mexico, providing HPV testing for the CC screening population. The national coverage of these HPV tests was 10% [18]. While HPV testing was available through these new laboratories, the CC screening program guidelines still recommended Pap-smear cytology as the primary screening test for women younger than 35 years of age; these patients are not screened for HPV infection [3]. For women over 35 years of age, HPV hybrid capture (HC) test screening is performed, and HPV-positive women are referred to Pap and colposcopy. The disadvantage of this strategy is the low sensitivity of the Pap and that not all areas of the country have access to colposcopy and HPV HC test. 

Genotyping of HR-HPV has shown the potential for facilitating cancer risk stratification of HPV-positive women [19]. In Mexico, in 2008, health institutions of the National Health System incorporated the hybrid capture (HC) test as a screening procedure for HPV detection in women aged 35 to 64 years [20]. However, the HC test only detects HR-HPV infection, not the HPV type. 

Serial type-specific HPV viral load measuring allows differentiation between regressing cervical lesions and serial virion productive transient infections [21]. HPV viral load in serial measuring could predict cervical lesion progression [22]. The risk of CC and HSIL increases with the viral load, which could triage HR-HPV-positive women [23,24]. 

The goal of this follow-up hospital case study was to determine if HPV types, multiple HPV infections, and viral load in women with gynecological alterations were associated with infection persistence and the cervical lesion grade.

## 2. Materials and Methods

### 2.1. Study Population

The study included 294 patients with gynecological alterations that had been referred to a colposcopy consultation at the Gynecology and Obstetrics Department of the Hospital Universitario “Dr. Jose Eleuterio González” of the Universidad Autónoma de Nuevo León (HU-UANL) in Monterrey, Nuevo Leon, Mexico. All of them agreed to participate in this study by signing an informed consent, previously approved on August 10, 2011 by the HU-UANL ethical committee (registration number BI11-002). After 6-12 months, HPV-positive patients were asked for a second cervical swab sample at a follow-up consultation. HPV-negative patients were considered as controls.

### 2.2. Sample Collection and DNA Extraction

Cervical cell samples were taken using a cytobrush (Colpoltre; Distribuidora de Insumos para la Salud Colpotre S.A. de C.V., Mexico City, Mexico), preserved in ThinPrep PreservCyt solution (ThinPrep Pap Test; Cytyc Corporation, Boxborough, MA, USA.), and stored at −70 °C until DNA extraction. Samples were collected from January 2014 to July 2016.

Samples were grouped according to Pap smear result: Normal, atypical squamous cells of undetermined significance (ASCUS), LSIL, HSIL, CC, and Unknown (when Pap smear result was unavailable).

DNA was extracted from cervical cells using the PureLink Genomic DNA kit from Invitrogen (Life Technologies, Carlsbad, CA, USA), following the manufacturer’s instructions. DNA quantity and quality were measured by spectrophotometry. DNA was stored at −20 °C.

### 2.3. HPV Genotyping and Viral Load

HPV was detected by PGMY 09/11 primers set using the *ß-globin* gene as an internal control [25,26]. PCR products were analyzed by 2% agarose gel electrophoresis, stained with ethidium bromide, and visualized in a UVP Model 2UV High-Performance Transilluminator (Upland, CA, USA). The PGMY system has been shown to detect a higher proportion of HR- and low risk (LR)-HPV types than other primer systems. Furthermore, it detects more HPV-positive samples than systems like MY 09/11.

HPV-positive samples were genotyped and quantified with Amplisens HPV HRC Genotype titer FRT kit (Ecoli, Bratislava, Slovak Republic) according to the manufacturer’s instructions in a 7500 Fast Real-Time PCR System (Thermo Scientific, Waltham, Massachusetts, USA). 

This kit is based on simultaneous real-time amplification (multiplex PCR) of DNA fragments of 14 HR-HPV types (HPV 16, 18, 31, 33, 35, 39, 45, 51, 52, 56, 58, 59, 66, and 68) and a DNA fragment of the *ß-globin* gene as an internal endogenous control, carried out in four separated reaction tubes. Each genotype is detected in a separate fluorescent channel (FAM, JOE, ROX, and Cy5) that allows its detection and quantification (a total of 16 qPCR probes are measured). Each genotype is detected in a separate fluorescent channel that makes it possible to determine the genotype and viral load. 

The quantitative analysis of HR-HPV DNA was based on the linear dependence between the initial concentration of DNA target in a test sample and the cycle threshold (Ct) (the cycle at which the fluorescence signal starts its exponential growth). Quantitative analysis was performed in the presence of DNA calibrators, and the Ct obtained values were used for calibration of HPV DNA (copies) quantity per 100,000 human cells. Results calculation and analysis were carried out automatically using the software Amplisens HPV HCR genotype-titer in Microsoft Excel format. The results were interpreted in logarithm HPV copies per 100,000 human cells, where less than 3 has a low clinical significance; 3–5 is clinically valuable and interpreted as a risk of dysplasia, and greater than five is clinically valuable and interpreted as highly suggestive of dysplasia. Test sensitivity is 1000 copies of HPV DNA per mL, with a linear range of HPV DNA measurement of 1000–100,000,000 HPV DNA copies per mL. The viral load results were log10 transformed for each HPV type (HPV per 100,000 human cells), to facilitate the interpretation of the results. The results are interpreted using a scale of log ≤3 (low clinical significance), 3–5 (clinically valuable, dysplasia cannot be excluded, risk of dysplasia), and >5 (clinically valuable, of increased value, dysplasia is highly suggestive). The kit was developed by Federal Budget Institute of Science “Central Research Institute for Epidemiology”; it was used following the manufacturer’s instructions and according to their clinical reports [27].

### 2.4. Follow-Up Samples

A second cervical sample was collected from HR-HPV-positive women after 6–12 months, and HPV genotyping and viral load measurements were taken for these.

### 2.5. Statistical Analysis

For the association of cytology category (Pap smear results: normal, ASCUS, HSIL, and LSIL) with HPV types, (14 types), multiple HPV infections, and viral load (high or low) (high: >3 log HPV copies/10^5^ cells, low: ≤3 log HPV copies/10^5^ cells), three contingency tables were used. The Kruskal-Wallis test was used to compare the Pap smear classification with the viral load (log 10 transformed) (HPV copies/ 10^5^ cells) for each HPV type. A *p*-value of less than 0.05 was considered significant.

## 3. Results

### 3.1. Sample Collection

We analyzed cervical samples and questionnaire answers from 294 patients (Table 1, Tables S1 and S2). Of these, 178 (60.5%) were HR-HPV-positive. HPV-positive patients had a sexual debut at an earlier median age than HPV-negative patients (*p* = 0.019). The number of sexual partners was not associated with HPV status. Only 3.4% of the study population had received an HPV vaccination; hence, vaccine efficacy could not be determined.

### 3.2. Pap Smears and HPV Genotyping at the First Consultation

**Figure 1:**
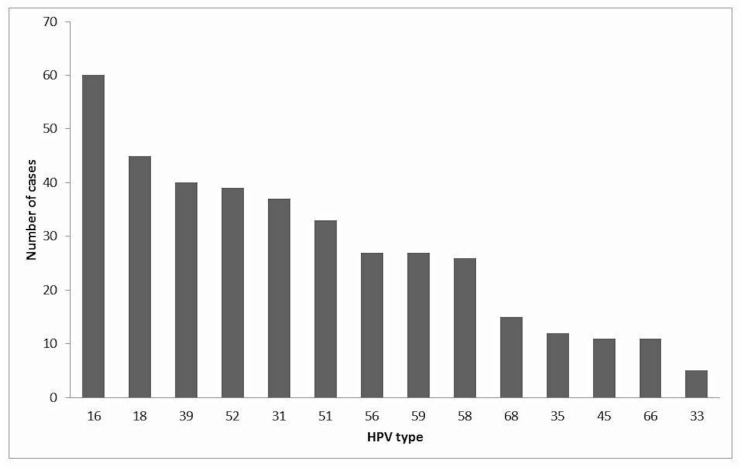
HPV-type prevalence found in our study group at the first consultation. We identified 378 viral sequences in 178 HPV-positive patients, of which 105 had multiple infections. The most frequent HPVs were 16, 18, 39, and 52.

From the 178 HPV-positive women, we found 48 (27%) women that had their first cytology sample with normal cytology result, 10 with an ASCUS (5.6%), 57 LSIL (32%), 47 HSIL (26.4%), and 3 CC (1.7%) (Table 2). The predominant HPV type was 16 (33.7%), followed by 18 (25.3%) and 39 (22.5%) (Table 3, Figure 1). HPV 18 and 16 were the most prevalent in normal cytology, LSIL, and HSIL samples (>20%). HPV 52 was only found in normal cytology and LSIL. The most prevalent HPV types and the association of these HPV types with multiple HPV infections are shown in Table 3.

We validated 54 of the HR-HPV single infected samples, using PGMY 09/11 PCR and then using capillary electrophoresis sequencing, which has been the gold standard method for viral detection testing to detect HPV-positive samples. We confirmed only single HR-HPV infections in those samples due to the nature of the Sanger sequencing chemistry. The Sanger sequencing of the partial amplified L1 gene region identifies single HPV infection and/or the most dominant genotype in the specimen. Sanger sequencing using PGMY 09/11 primers is useful only in single-type infections since it is impossible to obtain clean sequences in coinfections.

### 3.3. Multiple HPV Infections at the First Consultation

We found that 105 (59%) of the HPV-positive patients had multiple HPV infections (Table 2). The HSIL and the normal groups had the highest percentages of multiple HPV infections (62.5% and 62.1%, respectively), compared to the LSIL group (55.4%). We did not find multiple HPV infections in the ASCUS group. In LSIL, only HPV 16 and 18 showed a statistically significant association with multiple infections, while in HSIL, only HPV 56 was associated with multiple infections. Only statistical association of HPV 16 and 18 with multiple infections in LSIL and HPV 56 in HSIL was found.

We performed a chi-squared test to analyze the association between HR-HPV type and multiple HPV infections. We found a significant association between most of the HPV types, except HPV 33, 35, 45, 58 and 66, and multiple HPV infections. 

The most frequents multiple HPV infections found in the samples at the first consultation were 16-18, 18-51 and 18-52 (Table 4 and Table S3).

### 3.4. HPV Viral Load

**Figure 2:**
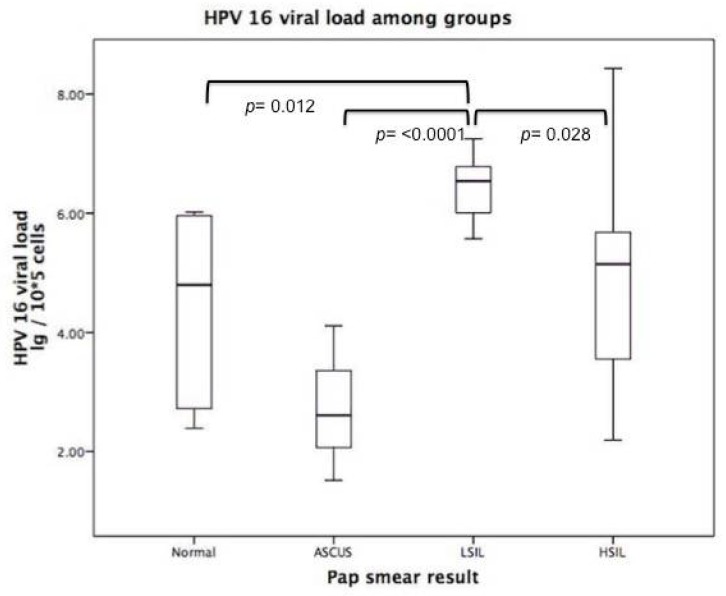
HPV 16 viral load at first consultation. In patients with low-grade squamous intraepithelial lesions (LSIL), a high viral load was found. The most heterogeneous group was that of patients with high-grade squamous intraepithelial lesions (HSIL).

Viral load was classified into high and low groups (see Section 2). We found associations between HPV 18, 51, and 56 and high viral loads (*p*-values of 0.012, 0.034, and 0.005, respectively). This result was independent of the Pap smear result (chi-squared contingency test *p* = 0.625). When we classified the samples by Pap smear result, we found that HPV 16 had a higher viral load in the LSIL and HSIL groups (Kruskal–Wallis test) (Figure 2). There was no significant difference between viral load of HPV 52 and the Pap smear groups (*p* = 0.253); significant difference was also not found when we analyzed HPV 18 (*p* = 0.312).

### 3.5. HPV-Positive Patients Follow-Up

We collected a second cervical sample from 81 HPV-positive patients. In these, we identified 31 (38.3%) samples with a persistent HPV infection, of which 16 (52 %) showed multiple HPV types. The persistent infection had the same HPV type in 18 samples. The most frequent HPV types in the 31 follow-up HPV-positive samples were 59, 56, 39, 52, and 16. Only HPV 39 had a statistically significant association with multiple HPV infections in the follow-up samples (*p* = 0.009). 

Seventeen (54.8%) of 31 samples that had multiple HPV infections at the first consultation had a persistent HPV infection at the second consultation, compared to the samples that clarified the HPV infection (*p* = 0.001) (Table S4).

We did not find any significant difference between the persistent HPV types, but we did find a significant and considerable difference (*p* < 0.001) in the HPV viral loads from the samples with a persistent infection (mean = 4.13, SE = 0.52) and the samples from patients who cleared the infection (mean = 1.64, SE = 0.39). 

## 4. Discussion

Epidemiologic HPV studies have helped to understand the prevalence and diversity of HPV in the population, and this is the first step in tailoring screening and prevention strategies to reduce cost and increase effectiveness of CC prevention efforts. 

Worldwide, the most prevalent HPV types are HPV 16, 18, and 52 [28]. In our study, based solely on participants presenting with gynecological alterations in Mexico, we found 14 different HR-HPV types, with HPV 16, 18, and 39 being the most prevalent. In two previous studies in Northeastern Mexico, HPV 59, 52, and 16 were previously reported as the most prevalent in normal cytology [14,29]. According to their pap smear result, we found that the most prevalent HPV types in LSIL were 18, 52, and 16, while 16, 39, and 18 were more frequent in HSIL [29]. In the previous study in Northeast Mexico, where a linear array method was used, the most prevalent HPV types in LSIL were 16 and 58 and in HSIL were 16 and 31. Studies conducted in the Mexican population have shown differences in the prevalence of viral genotypes depending on the geographical area. For example, a study conducted in the states of Aguascalientes, Jalisco, and Zacatecas found that HPV 51 and 16 were the most prevalent genotypes [30]. A study based on the population in the state of Tlaxcala found that HPV types 16 and 18 had the highest prevalence [31]. These results highlight the importance of HPV genotype prevalence and distribution in different regions of Mexico [7,14,15,16]. Methodological strategy, studied population, and selection criteria are some of the drawbacks of comparing existing studies. 

We detected 48 HR-HPV-positive samples with apparent normal cytology, where 62.1% had multiple HR-HPV infections and 63.8% had a high viral load. A high viral load has previously been associated with the risk of persistent HPV infection [32]. We found that the patients with a persistent infection had a higher viral load at the time of the first sample collection, a finding that has been previously reported as a risk factor for persistent infection in other studies [33]. 

Few HPV studies properly follow up on patients, limiting epidemiological studies on HPV infection persistence and viral load changes during disease progression. The studies that do employ a follow-up schema can analyze not only prevalence and distribution, but also genotype persistence, viral load over time, and progression behavior of both single HPV infections and multiple HPV infections. 

Along with sociocultural factors, ethnicity has shown to be an influence on the prevalence of HPV. For example, a study conducted in the United States that included 4080 females of which 29.7% were African-Americans, 25.6% were Mexican-Americans, 8.9% were Hispanics, and 35.8% were White, considering pre-vaccine years (2003–2006), vaccine-type HPV, and late postlicensure years (2011–2014), there was a significant decrease in prevalence in the first two groups but not in Mexican-Americans [34].

We found that 59% of the HPV-positive samples had multiple HR-HPV infections. Other studies reported multiple HPV infections: 68.2% in HSIL [35] and 13.2% in FFPE samples [36]. Another study reported that 38.2% of the cases had a multiple HPV infections, which is lower than the value reported in our study (59%). Also, in this study, multiple HR-HPV infections did not correlate to an increased risk of CIN1+ compared to single HR-HPV infections [37]. Another report in a follow-up study showed that high viral load did not predict an increased risk of HSIL and CIN3+. This observation was corroborated by our findings in that a high viral load of HPV 16 was significantly different in LSIL compared to other groups [38]. Recent studies have shown a decrease in multiple HPV infections in HSIL cases compared to LSIL, unlike our study, where we found 55% multiple HPV infections in LSIL versus 62% in HSIL; in this Brazilian study, they found 58% multiple HPV infections in LSIL and 55% in HSIL cases [39]. Considering that our method is unable to detect LR-HPV, we cannot discard samples that might also have multiple HPV infections with an LR-HPV. In HSIL, 62.5% of cases presented multiple HR-HPV infections, and this result was statistically significant. According to a meta-analysis considering the Latin American and Caribbean population, multiple HPV infections were found in 16.8% of HSIL samples and 12.6% of CC [13]. 

On the other hand, a recent study conducted in Central Mexico reported 26.3% of multiple HPV infections in the overall group. We found 59% of multiple HPV infections in the overall group of Northeastern México. Our results found a higher prevalence of multiple HPV infections than those previous reports in Mexico and Latin America mentioned before. It is important to notice that, in our study, most of the patients had a clinical history of sexually transmitted diseases (STDs).

Multiple HPV infections have been previously associated with persistent HPV infections [39]; in our study, 55% of the patients who had a multiple HPV infections had a persistent infection six months to one year after the first sample was taken. In this study, we did not identify which of the viruses in the multiple-HPV-infected patients were transcriptionally active. Detection of viral oncoproteins expression (such as E6 and E7) by viral type-specific real-time PCR could indicate if there is one or more transcriptionally active virus in the co-infected samples.

We found a significant difference among Pap smear groups (cytology category severity) and HPV 16 viral load, increased in LSIL, which is consistent with findings in the Danish population [40], though variance suggests these findings are either due to chronological differences in the study design or region-specific factors unaccounted for in this type of study [41]. Despite the wide ranges found in our study for HPV 16 viral load in the HSIL group, LSIL had a significantly higher viral load. While associations between cytologically identified lesions and viral load may differ across studies, viral load has been identified as a risk factor in CC development with consistency across populations [42,43,44,45]. There are also data to suggest that the risk of CIN1+ could have a type-dependent association [46]. 

Patients with a normal cytology result had a lower HPV 16 viral load than those with an LSIL result (p = 0.012). This might indicate that there is an active HPV replication, which is consistent with other studies where viral load decreased with an increasing grade of the cervical lesion but increased in cancer cases [47]. Their data support the concept of viral events in cervical carcinogenesis where initially high viral loads could increase the probability of integration. After selection of the integrated cell clones that progress towards cancer, the viral load could be reduced again [47].

Detecting and treating HPV infections will help reduce CC progression. Cervical intraepithelial neoplasias are treated with cervical conization surgery, LEEP, or cryotherapy to reduce CC development. Patients included in this study with an abnormal cytology result received treatment according to its clinical status, and most of them had a clinical history of STDs. Furthermore, these patients were deemed a risk group from the colposcopy consultation and had gynecological alterations previously detected at the general gynecology and obstetrics consultation.

In the follow-up study, we found that 38.3% of the HPV-positive women had persistent infections. Other studies reported a prevalence from 10% to 40.9% [35,48,49] of persistent infections. These previous studies detected HPV infection a year after the first sample was taken. Pirtea et al. carried out a follow-up study where they took samples with a lapse of six months or less and found 41% persistent infections, similar to our study [35]. This persistence was reduced to 20% after one year of follow-up. In our study, the follow-up sample was taken with a lapse between six months and one year, suggesting that the persistence observed in our population was not due to the time of the follow-up.

In a study performed in Northeastern Mexico in 2002 [14] 19.4% of HPV persistence was found, differing from our results (38.3%). They reported an open screening study, while our population was sampled from patients with gynecological alterations. The most persistent HPV types for those 3-year follow-up samples were 18 and 16, while in our study the most persistent HPV types were 59 and 56. We defined persistence as HPV positivity at a minimum of two time-points [50], established by the International Agency for Research on Cancer (IARC) as a minimum of 4 months but up to 5-7 years [51]. Even though it has also been established that persistence is based on how long the infection lasts [52], and type-specific persistence has been shown [53], for this purpose, we did not consider “reinfection” with the same or different viral type [54] due to the lack of sexual activity information. It is difficult to estimate if it is a “reinfection” (the same virus strain) or if it is a different one regardless of being the same HPV type and subtype; only next-generation sequencing would provide this information due to the lineage multiple infections that are not detected with the usual methods [55].

This study allowed us to analyze viral factors associated with persistence since we carried out a follow-up study in patients who attended the colposcopy clinic. Follow-up studies allow a more accurate assessment of viral resistance. We evaluated patients at high risk of developing CC in the colposcopy consultation. All these patients had a history of previous sexually transmitted diseases. The use of visual inspection with acetic acid (VIA) to identify cancerous lesions, the detection of high-risk HPV, and the measurement of viral loads, combined with the use of colposcopy by a specialized physician, allowed us to evaluate patients in integrally and comprehensively. HPV-positive and Pap-positive patients should be confirmed using colposcopic VIA, since this technique improves the sensitivity for the detection of CC. Colposcopic VIA has the potential to be a CC screening tool, especially in low-income countries [56].

The HPV vaccination in Mexico began in 2009 [57]. The bivalent vaccine (Cervarix) was implemented with a three-dose schedule (0, 6, and 60 months) [57]. At present, a scenario of universal vaccination coverage against HPV in Mexico is not viable, given the high costs of the vaccine [58]. The most feasible cost-effective option was to follow a selective vaccination strategy, where women living in marginalized areas and with less access to health services have priority to receive the vaccine [58]. Currently, the coverage of the HPV vaccine in Mexico is 40% [59]. The goal is to reach a national coverage greater than 70% in girls aged 12 to 16 years. In our study, only 3.4% of the women had received the HPV vaccine. One patient vaccinated against HPV 16/18 had a multiple HPV infection with five different HPV types, including HPV 18. However, this patient only received a single dose of the vaccine. The other two vaccinated patients that had a single dose of the vaccine tested HPV-negative. In Mexico, vaccination of girls aged 9 to 11 years old began in 2009, thus most of the patients included in this study were not vaccinated. The effectiveness of the vaccine could not be analyzed in this study. HPV genotype detection in the different regions of Mexico provides valuable information to assess the impact that the HPV vaccine could have if the vaccination coverage was greater than 70%.

To decrease the mortality rate of CC in Mexico, we must expand coverage of the Pap, improve quality control programs for cytopathology laboratories, integrate screening strategies (combined Pap smear, HPV testing, and HPV vaccination), improve colposcopy coverage, and implement new health education strategies regarding CC [4,60].

## 5. Conclusions

The persistence of HPV lesions is among the most important risk factors for the progression of CC and perhaps the first for progression to lesions. We identified HPV 16, 18, and 39 as the most prevalent HPV types in cervical cytology in a group of Mexican women with gynecological alterations. HPV 16, 18, 31, 35, 39, 45, 52, 56, and 59 were associated with high viral loads, while most of the HR-HPV types were associated with multiple HPV infections. HPV 16 viral load was higher in patients who had LSIL and HSIL. The 54.8% of the patients with multiple HPV infections were HPV-positive at the second consultation. A higher viral load was found in the first consultation samples of the patients who showed an HPV infection after six months to a year follow-up. The follow-up study provides more evidence that multiple HPV infections and high viral loads can be valuable markers for identifying patients at high risk of having persistent infections and developing CC.

## Figures and Tables

**Table 1:** Gynecological characteristics.

Clinical Variable	Median	*p*-Value
Age		
HPV positive	34.90	0.169
HPV negative	37.05	
Number of sexual contacts		
HPV positive	2.95	0.586
HPV negative	2.90	
Age of first sexual contact		
HPV positive	17.88	0.032
HPV negative	18.66	
Treatment received*	cases	%
Cervical conization surgery	31	17.4
LEEP	17	9.6
Hysterectomy	20	11.2
Cryotherapy	11	6.2

* Only the patients who had this information are included. Loop electrosurgical excision procedure (LEEP).

**Table 2:** Sample Pap smear groups and cases of multiple human papillomavirus (HPV) infections.

Pap Smear Result	Total Cases per Group	Total Cases of Multiple HPV Infections		*p*-Value
		Number of Cases	% of Cases	
Normal	48	36	75.0	0.2431
ASCUS	10	0	0.0	0.0147
LSIL	57	31	54.3	0.7598
HSIL	47	30	63.8	0.7968
CC	3	1	33.3	1.0000
Not available	13	-	-	
Overall	178	105	59.0	

**Table 3:** HPV prevalence and its association to multiple HPV infections among Pap smear groups at the first consultation.

Patients Groups HPV Type	# cases	Multiple Infection # Cases	%	*p*-Value Chi
Overall HPV types (top)				
16	60	47	78.3	0.0001
18	45	38	84.4	0.0001
39	40	34	85.0	0.0001
52	39	32	82.1	0.001
51	33	26	78.8	0.011
Normal				
52	18	16	88.9	0.009
18	17	16	94.1	0.002
16	16	15	93.8	0.005
39	14	12	85.7	0.062
51	13	10	76.9	0.338
LSIL				
18	15	12	80.0	0.034
52	13	9	69.2	0.345
16	12	11	91.7	0.007
51	12	9	75.0	0.191
39	11	9	81.8	0.880
58	11	8	72.7	0.312
HSIL				
16	23	17	73.9	0.145
39	12	10	83.3	0.167
18	11	9	81.8	0.171
31	11	9	81.8	0.171
56	9	9	100.0	0.018

**Table 4:** Most frequent high-risk human papillomavirus (HR-HPV) types found in multiple infections at the first consultation.

Most Frequent HR-HPV Multiple Infections	
HPV types	Cases
16, 18	15
18, 51	13
18, 52	12
39, 51	12
39, 52	12
Others	41

## Supplementary Materials

The following are available online at https://www.mdpi.com/1999-4915/12/4/380/s1, Table S1: Sociodemographic parameters, Table S2: Gynecological and obstetric characteristics, Table S3: HR- HPV-types in cases with multiple infections at the first consultation, Table S4: Comparison of the HR-HPV types found in the persistent HPV- infected samples in the follow-up study.
