# Supplementary material for: Multiple HPV Infections and Viral Load Association in Persistent Cervical Lesions in Mexican Women
Source: Viruses. 2020 Mar 31;12(4):380. doi: 10.3390/v12040380 (PMC7232502; doi:10.3390/v12040380)
Supplement: Supplementary file 1 [file viruses-12-00380-s001.pdf]

Table S1. Sociodemographic parameters.

Patients sociodemographic characteristics		
**n = 294**	**HPV+ n = 178**	**HPV− n = 116**
Age	35 (±12)	37 (±12)
Place of birth (Northeast region)	93%	89%
Profession (Housewife)	47%	60%
Marital status (Married)	43%	44%
Scholarship	46%	36%
Family history of cancer	43%	36%
Comorbidity	14%	16%
Alcohol consumption	30%	27%
Smoking history	34%	27%
BMI (normal 19%-25%)	39%	25%

Table S2. Gynecological and obstetric characteristics.

Gynecological and obstetric background		
	**HPV+**	**HPV−**
Menarche age	13 (±1.6)	13 (±1.6)
Active sex life initiation (<18 years old) $p = 0.019$	64%	56%
Sexual partners (>1)	77%	62%
Barrier contraceptive methods	33%	20%
STD	54%	33%
HPV vaccinated	3%	1%
First Pap smear after first sexual contact	6 years	6 years

Table S3. HR-HPV-types in cases with multiple infections at the first consultation.

Cases	HR-HPV Multiple Infections	Cases	HR-HPV Multiple Infections
**1**	16, 18	1	18, 45
**1**	16, 18, 33	1	18, 51
**2**	16, 18, 39, 52, 51	2	18, 51, 52
**2**	16, 18, 51	3	18, 52
**1**	16, 18, 51, 52	2	18, 58
**2**	16, 18, 59	1	31, 52, 59
**1**	16, 18, 59, 51	1	31, 35, 39, 52
**1**	16, 18, 59, 66	1	31, 39, 45, 59
**1**	16, 18, 59, 68	1	31, 39, 52, 56, 59
**2**	16, 18, 68	2	31, 52, 56
**1**	16, 18, 68, 56, 58	1	31, 58
**4**	16, 31	1	31, 56, 59
**1**	16, 31, 18	1	35, 39, 51, 52, 56, 68,
**1**	16, 31, 18, 35	1	35, 39, 58
**1**	16, 31, 18, 59	1	39, 56, 59, 51, 52
**1**	16, 31, 18, 66	1	39, 51
**1**	16, 31, 39, 52	1	39, 51, 52, 66
**1**	16, 31, 39, 59, 68, 56, 52	2	39, 51, 59
**1**	16, 31, 45	1	39, 52, 56, 68
**1**	16, 31, 59, 52	2	39, 56
**1**	16, 35	1	39, 58
**5**	16, 39	1	39, 59
**1**	16, 39, 45, 59	1	39, 59, 56, 51
**1**	16, 45	2	35, 52
**4**	16, 51	1	45, 58
**2**	16, 56	2	51, 59
**6**	16, 58	1	52, 56
**1**	16, 58, 51	1	52, 56, 68
**1**	18, 31, 52	2	52, 58
**1**	18, 35, 66	1	52, 59
**1**	18, 39	1	56, 58
**1**	18, 31, 39, 33, 56	1	56, 59
**1**	18, 39, 33, 52	1	56, 68
**1**	18, 39, 45, 52, 58, 56	1	58, 68
**1**	18, 39, 51	1	59, 68
**1**	18, 39, 52, 51	1	33, 45, 68
**1**	18, 39, 59, 51	1	33, 58, 68
**1**	18, 39, 59, 56	1	35, 66

Table S4. Comparison of the HR-HPV types found in the persistent HPV-infected samples in the follow-up study.

Sample #	HPV type in first consultation	HPV type in second consultation	Persistent HPV	New HPV type acquired	HPV type eliminated
1	16	16, 31, 18, 59	16	31, 18, 59	-
2	59, 56	39, 59, 56	59, 56	39	-
3	31, 39, 35, 52	31, 39, 59, 52	31, 39, 52	0	35
4	16, 45, 35	16, 66	16	66	35, 45
5	18	31	0	31	18
6	31, 18, 52	39, 59, 56, 52	52	39, 59, 56	31, 18
7	59	59	59	-	-
8	68, 56, 52	39, 56	56	39	68, 52
9	66	59	-	59	66
10	39, 35, 68, 56, 52, 51	59, 68	68	59	39, 35, 56, 52, 51
11	18	18	18	-	-
12	45	56	-	56	45
13	16	16, 59	16	59	-
14	16, 18, 59	59	59	-	16, 18
15	31, 56, 52	39, 31, 52, 56	52, 56, 31	39	-
16	52	39, 59, 52, 56	52	39, 59, 56	-
17	45	51	-	51	45
18	16	58	-	58	16
19	56	39, 56	56	39	-
20	16, 18, 68	16, 58	16	58	68
21	39, 59, 51	16, 58	-	16, 58	39, 59, 51
22	39, 58, 52, 45, 18, 56	39, 56	39, 56	-	58, 52, 45, 18
23	33, 68, 58	68, 58	68, 58	-	33
24	18, 39	51	-	51	18, 39
25	18, 39, 33, 52	33, 52	33, 52	-	18, 39
26	52	52	52	-	-
27	16	16, 31	16	31	-
28	56	59	-	59	56
29	51	56	-	56	51
30	16, 31, 39, 59, 68, 56, 52	31	31	-	16, 39, 59, 68, 56, 52
31	16, 31, 59, 52	16, 31, 59, 68, 58, 52	16, 31, 59, 52	68, 58	-
